# Choroidal melanoma metastasizing to maxillofacial bones

**DOI:** 10.1186/1477-7819-5-30

**Published:** 2007-03-08

**Authors:** Manoj Pandey, Om Prakash, Anitha Mathews, Nileena Nayak, Krishnankutty Ramachandran

**Affiliations:** 1 Division of Surgical Oncology, Regional Cancer Centre, Trivandrum, Kerala, India; 2 Pathology, Regional Cancer Centre, Trivandrum, Kerala, India; 3 Imagiology, Regional Cancer Centre, Trivandrum, Kerala, India; 4 Surgical Oncology, Institute of Medical Sciences, Banaras Hindu University, Varanasi, India; 5 Oral and Maxillofacial Surgery, Government Dental College, Thiruvananthapuram, India

## Abstract

**Background:**

Melanomas are malignant neoplasm of melanocytic origin, commonly seen on skin and various mucous membranes. Melanomas are the commonest intraocular malignant tumour in the adults.

**Case presentation:**

A 50-year-old female presented with complains of painless progressive swelling in right cheek region of two months duration. Examination revealed a 6 × 4 cm bony hard swelling in right zygomatic region near and below lateral canthus of right eye with loss of vision. Investigations revealed it to be a choroidal melanoma metastatising to the zygomatic bone. Patient was successfully treated by surgery.

**Conclusion:**

Choroidal melanoma, which commonly metastasizes to liver and lungs, never involves the lymph nodes and metastasis to facial bones is rare. Here we report a case of choroidal melanoma metastasizing to maxillofacial bones.

## Background

Choroidal melanoma is the most common primary intraocular malignant tumour [1]. They arise from uveal melanocytes residing in the uveal stroma and originating from the neural crest [2]. Choridal melanomas are quite different from their cutaneous counterparts with regards to presentation, metastatic pattern and treatment. The incidence of intraocular melanoma is less than 1 per 100000 [3]. Spread to the liver is the most frequent while metastases [4] to other sites (lung [5], heart [6,7], gastrointestinal tract, lymph nodes [8], pancreas, skin, central nervous system [9], bones, spleen, adrenal [10], kidneys, ovaries [11], thyroid [12], contralateral choroids [13] breast [14]) generally occur in association with liver metastases [2]. Lymphatic spread has not been demonstrated, consistent with the absence of lymphatics in the choroid. To our knowledge choroidal melanoma metastasizing to the maxillofacial bones and presented with malar swelling is never been reported before.

## Case presentation

**Figure 1:**
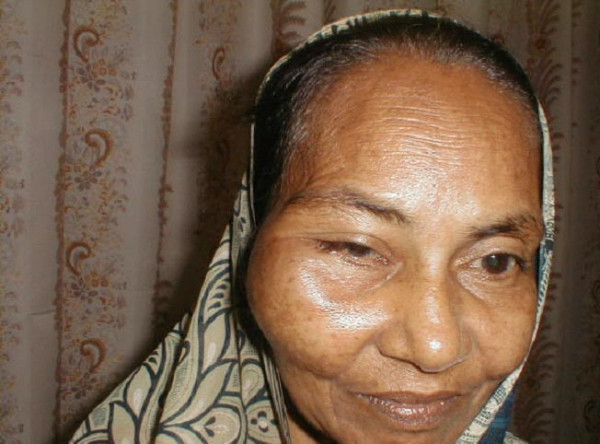
Clinical photograph showing swelling in right zygomatic region.

**Figure 2:**
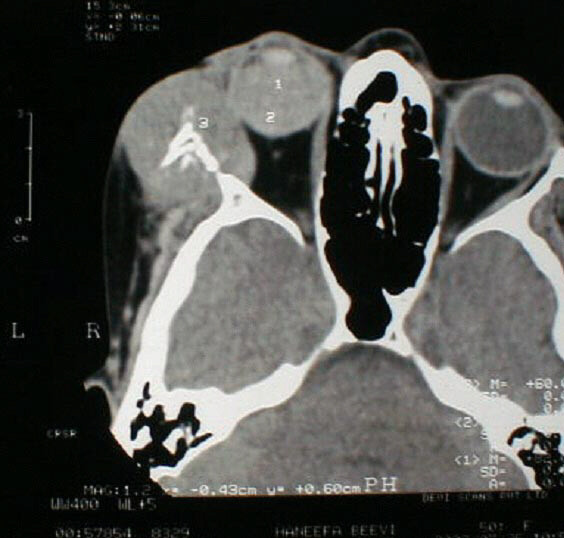
Computed tomographic scan showing two separate lesions one in right orbit and other in right zygoma, zygomatic process of maxilla.

**Figure 3:**
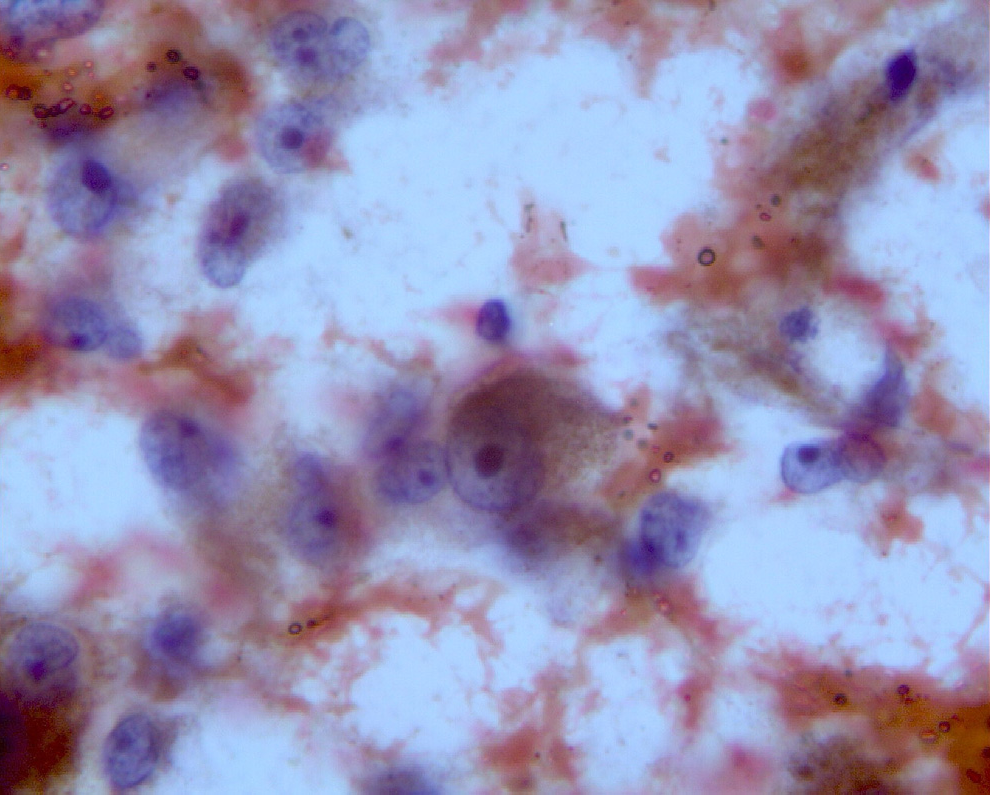
Photomicrograph of FNAC showing typical polygonal and spindle cells with moderate amount of cytoplasm and vesicular nuclei with nucleoli (Haematoxylin & Eosin original magnification ×100).

**Figure 4:**
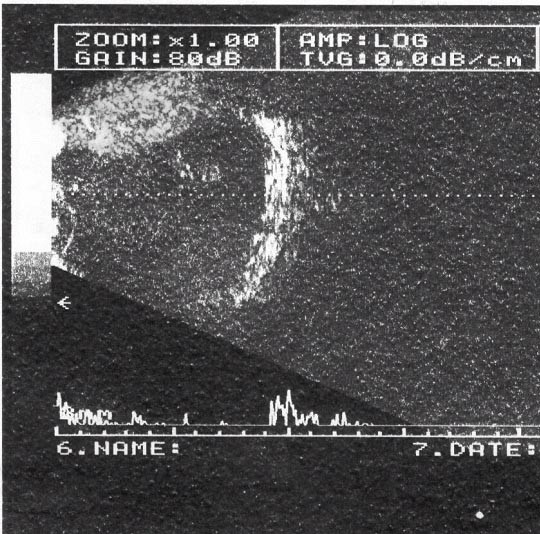
B-scan of the right eye showing an organized mass within the vitreous cavity suggestive of vitreous haemorrhage.

A 50-year-old female presented with complains of painless progressive swelling in right cheek region of two months duration. Examination revealed a 6 × 4 cm bony hard swelling in right zygomatic region near and below lateral canthus of right eye (Figure 1) Roentgenogram of the paranasal sinuses showed a lesion arising from right maxilla and right zygoma. Computerized tomographic (CT) scan revealed two separate masses one in right orbit and other infiltrating zygoma and maxilla (Figure 2), radiographic picture was suggestive of osteogenic sarcoma or Ewing's sarcoma. A fine needle aspiration was carried out that showed typical polygonal and spindle cells with moderate amount of cytoplasm and vesicular nuclei with nucleoli, many with intracellular pigment; seen singly and clusters, the picture was suggestive of malignant melanoma (Figure 3). The ophthalmologic consultation revealed that there was no useful vision in the right eye. A B-scan of the right eye showed an organized mass within the vitreous cavity indicating vitreous haemorrhage (figure 4). A detailed clinical examination failed to show any other melanocytic lesion on skin and other mucus membranes.

**Figure 5:**
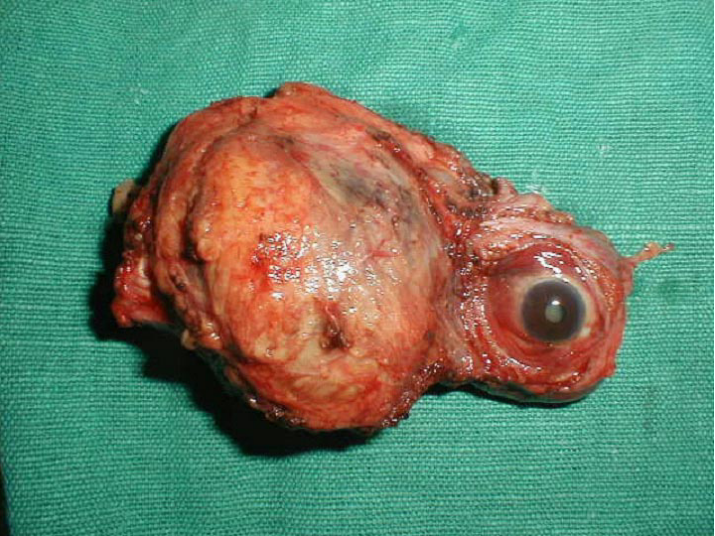
Photograph showing resected tumour with eyeball exenteration.

**Figure 6:**
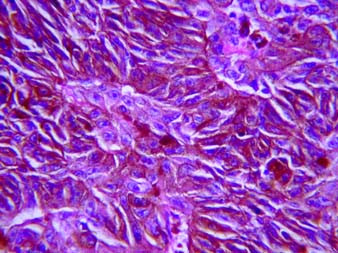
Photomicrograph showing tumor composed of sheets, nests and cords of polygonal cells and vesicular nuclei. Prominent nucleoli and eosonophilic cytoplasm containing abundant melanin pigment (Hematoxylin & Eosin original magnification ×40).

With a provisional diagnosis of melanoma of the maxilla and zygoma with vitreous haemorrhage and no useful vision in right eye a wide excision of the lesion including anterolateral maxillectomy, zygomectomy, and removal of superolateral wall and floor of orbit with en bloc exenteration of the right eyeball was carried out (Figure 5). Histopathological examination of the resected eyeball specimen showed a neoplasm in choroid, filling posterior chamber with no extraoccular extensions, composed of sheets, nests and cords of polygonal cells and vesicular nuclei. Prominent nucleoli and eosinophilic cytoplasm containing abundant melanin pigment were noted (figure 6). Similar tumours were seen in the extra ocular soft tissue and resected maxilla and zygomatic bones. A final diagnosis of choroidal melanoma metastasizing to maxillofacial bones was made. Differential diagnosis of primary bone melanoma with metastasis to choroids was kept.

Surgical excision was followed by 40 Gy./15 fractions of radiotherapy to right face using lateral portal. Patient is on regular follow-up two years after surgery and is free of any local or systemic recurrences.

## Discussion

Malignant neoplasms of melanocytic origin, malignant melanoma is rare in soft tissue and bone. In eyes it may involve choroids, ciliary body, retina or conjuctiva [15-17]. Choroidal melanomas are commonly asymptomatic and are often discovered during routine ophthalmic examination however; in some cases it may produce symptoms like loss of vision, photopsias and visual field defects [18]. Intraosseous malignant melanoma on the other hand is extremely rare [15].

Diagnosis of choroidal melanoma is based on ophthalmoscopy, fluorescent angiography, scleral transillumination, B-scan ultrasonography, and sequential diagnostic examination [19]. Tumour markers like TA-90 IC, Melan A, HMB-45, S-100 add to diagnostic accuracy [20]. The classic signs of melanoma seen with B-mode ultrasonography include an acoustically silent zone within the melanoma, choroidal exacavation and acoustic shadowing of the orbit [21].

The treatment of choroidal melanoma includes local radiation with charged particles or epsiscleral plaque brachytherapy, tumour resection, enucleation and hyperthermia [22]. Transpapillary thermotherapy (TTT) is new treatment for small melanomas and is capable and causing necrosis of tumour up to 3.0 mm thickness [23]. Large melanomas (>10 mm in thickness or >1125 cm^2 ^in basal area) are usually managed with enucleation [24].

When malignant melanoma is encountered in the bone three possibilities are considered for its origin 1) skeletal metastasis 2) direct bony invasion 3) primary clear cell sarcoma. Out of these a skeletal metastasis of malignant melanoma is most frequent cause. The other organs involved in case of metastasis are lungs, liver and brain [15]. Clear cell sarcoma, or malignant melanoma of soft parts can invade the bone along the course of attachments of tendons and ligaments; however, this pattern of direct invasion has been considered to be exceedingly rare [17,25]. Primary clear cell carcinoma arising from bone itself is also very rare. Direct bony invasion did not occur in this case since the lesions were 2 separate masses demonstrated on CT scan.

Malignant melanoma of bone has a variable and unpredictable course with high rate of recurrence after excision. Most authors recommend complete radical excision of the tumour along with excision of regional lymph nodes. Adjuvant chemotherapy and radiotherapy may be of benefit as well. Despite treatment, the overall prognosis is poor and death usually access because of wide spread dissemination of disease [26].

## Conclusion

Unlike cutaneous melanoma, malignant melanoma of the choroids does not metastasize to lymph nodes because they do not have lymphatic drainage however, they normally metastasize to liver, lungs or brain. Unusual metastasis poses a diagnostic and therapeutic challenge. The uniqueness of the case reported is its metastasis to maxillofacial bones (maxilla and zygoma) without involvement of lungs, liver or brain, which to our knowledge has not been documented earlier.

## Competing interests

The author(s) declare that they have no competing interests.

## Authors' contributions

**MP**: conceived the idea, participated in manuscript preparation and edited the final version

**OP**: wrote the draft manuscript and did the literature search

**NN **and **AM**: performed the pathology and prepared the photomicrograph

**KR**: performed the radiology and contributed to drafting the manuscript.
